# A Hybrid ISSA-XGBoost Model for Predicting Wellbore Leakage

**DOI:** 10.3390/s26113526

**Published:** 2026-06-02

**Authors:** Kai Bai, Jiaqi Chen, Senlin Yin, Chaojie Wei, Yuzhou Yan, Junjie Liu

**Affiliations:** 1 School of Computer Science, Yangtze University, Jingzhou 434023, China; 2024720761@yangtzeu.edu.cn (J.C.); 2024710744@yangtzeu.edu.cn (C.W.); 2024710759@yangtzeu.edu.cn (Y.Y.); 2024710735@yangtzeu.edu.cn (J.L.); 2 Artificial Intelligence Research Platform, Yangtze University, Jingzhou 434023, China; 3 Hubei Key Laboratory of Drilling and Production Engineering for Oil and Gas, Yangtze University, Wuhan 430100, China; 4 Research Institute of Mud Logging Technology and Engineering, Yangtze University, Jingzhou 434023, China; yinsenlin@yangtzeu.edu.cn

**Keywords:** wellbore leakage prediction, sparrow search algorithm, gradient boosting decision tree, Sobol sequence, opposition-based learning, intelligent optimization

## Abstract

As critical underground engineering structures, wellbores may suffer complex structural deterioration and hidden safety hazards may be encountered during drilling. Multi-source sensor monitoring data provides an effective data basis for structural health perception and early warnings for wellbore structures at risk. The inherent diversity of formation conditions and the dynamic disturbances during drilling jointly lead to the differentiated presentation of drilling loss types, among which fractured, permeable, and vuggy losses are the most typical. This paper focuses on fractured wellbore leakage, regards wellbore leakage as an important structural failure form of underground drilling engineering structures. In-depth analysis and research on the structural deterioration mechanism of wellbore leakage were conducted, and we propose a wellbore leakage prediction method based on the improved sparrow search algorithm (ISSA) optimized gradient boosting decision tree (XGBoost). First, the Sobol sequence is adopted to replace the random initialization strategy, combined with the opposition-based learning mechanism; then, an adaptive Levy flight search mechanism is introduced to dynamically adjust the population ratio of discoverers and vigilantes; finally, intelligent optimization technologies are integrated to reconstruct the position update strategies of discoverers, followers, and vigilantes, enhancing the optimization adaptability of the algorithm. Relying on multi-field sensor monitoring datasets collected from actual drilling engineering, this paper compares the proposed model with wellbore leakage prediction models built by classical machine learning algorithms, and verifies its generalization ability on different datasets. Experimental data indicate that the improved algorithm exhibits significant advantages in optimization accuracy, enabling the proposed model to achieve an AUC improvement of 4.46%, along with accuracy (95.1%), precision (94.9%), recall (94.7%), and F1-score (94.2%). On this basis, the ISSA was applied to the hyperparameter optimization of XGBoost, constructing the ISSA-XGBoost prediction model. The method has high accuracy and good generalization ability in fractured wellbore leakage prediction, and it can realize intelligent health monitoring of underground wellbore structures, including early warnings. This study provides a reliable sensing data analysis scheme and technical support for structural health monitoring and hazard prevention in drilling engineering.

## 1. Introduction

Wellbore leakage is a pervasive and costly challenge in drilling engineering, particularly in complex formations with narrow mud-weight windows, such as fractured or pressure-depleted zones [1]. It causes extended non-productive time and excessive drilling fluid consumption, and severe complications like wellbore instability and blowouts, accounting for up to 20% of total drilling costs in extreme cases [2], and it affects 67.2% of wells in shale gas fields [3]. As a critical underground engineering structure, the wellbore is prone to structural deterioration and micro-fracture propagation under complex formation stress and drilling disturbance. Wellbore leakage is regarded as one of the most typical structural failure forms of subsurface drilling structures, which seriously endangers the structural health integrity of wellbore [4]. Accurate prediction and early warning of lost circulation are thus crucial for safe and economical drilling, making it a key research hotspot in petroleum engineering [5].

Traditional wellbore leakage prediction methods have inherent limitations in complex, less-explored areas. Comprehensive logging relies heavily on historical data and regional geological models, and insufficient prior data leads to reduced prediction accuracy [6]. Acoustic measurement technology solves the problem of accurate downhole liquid level identification after leakage, while noise logging can assist in identifying leakage layers, but both have adaptability bottlenecks under extreme conditions [7]. Conventional paddle flowmeters also suffer from poor accuracy due to drilling fluid oscillations [8]. Most traditional monitoring methods rely on single sensing equipment, which cannot capture the multi-physical field evolution characteristics of wellbore structural degradation. The insufficient utilization of multi-source synchronous sensing data restricts the real-time health perception capability of underground wellbore structures [9]. For better prediction performance, scholars have developed mechanism-based models. Chen et al. built a risk prediction model using fractured zone shale content as the key indicator, but it lacks generalization in new geological scenarios [10]. Ghalambor A et al. proposed a well-seismic integration evaluation system, which is highly dependent on data quality and ignores geological factor coupling [11]. In critically assessing the above methods, physics-based models offer interpretability but require real-time unavailable formation parameters; conventional ML relies on manual features and degrades under varying conditions; deep learning demands large labeled datasets and suffers hyperparameter sensitivity. None specifically address fractured wellbore leakage where multi-parameter nonlinear coupling dominates. Thus, unresolved gaps remain: lack of adaptive hyperparameter optimization, limited field validation using real fractured-reservoir data, and insufficient practical deployment discussion.

With the rapid advancement of artificial intelligence, machine learning (ML) methods have been increasingly applied in engineering anomaly early warning due to their superior capability in processing complex nonlinear data. In the field of lost circulation prediction, data-driven ML models have demonstrated remarkable potential compared to traditional methods. Gan et al. proposed a hybrid model combining support vector regression (SVR) with mutual information-based feature selection to improve prediction performance [12]. Liu Q et al. [13] developed a DNN-based model to determine real-time alarm thresholds, realizing accurate detection of abnormal mud loss by analyzing flow rate and mud volume data. Pang H W et al. constructed a multi-source data fusion framework integrating BP-LSTM and seismic attributes, capturing dynamic and static features from 55 logging and seismic parameters [14]. Additionally, Ding Z et al. reported that SVM, RF, and K-NN models based on surface mechanical parameters can achieve reliable leakage rate prediction with minimal downhole equipment dependency [15]. The widespread deployment of multi-type downhole sensors continuously acquires massive real-time drilling monitoring datas, providing a reliable data foundation for intelligent structural health monitoring of wellbores. Data-driven machine learning algorithms have become an effective technical means to mine implicit structural deterioration information from multi-sensor monitoring datasets [16]. Compared to recent state-of-the-art works, our ISSA-XGBoost offers better trade-offs between accuracy, interpretability, and computational efficiency for real-time field use.

The Extreme Gradient Boosting algorithm (XGBoost), proposed by Chen and Guestrin in 2016, is a highly efficient machine learning algorithm. It offers advantages such as excellent predictive accuracy, outstanding generalization capabilities, support for efficient parallel computing, and robust feature selection capabilities. It is particularly well suited for modeling tasks involving high-dimensional nonlinear data. Applying this algorithm to the petroleum drilling field enables precise prediction of wellbore leakage risks, holding significant theoretical and practical value for ensuring drilling safety, reducing engineering costs, and enhancing construction efficiency. Previous researchers [17] employed Whale Optimization Algorithm (WOA), Grey Wolf Optimizer (GWO), and Bayesian Optimization (BO) algorithms to optimize XGBoost hyperparameters, effectively improving model predictive performance. The Sparrow Search Algorithm (SSA), as a novel swarm intelligence optimization algorithm, constructs an optimization model based on the foraging and anti-predation behaviors of sparrow populations. Its unique hierarchical search mechanism and adaptive behavioral strategy enable it to demonstrate significant advantages in complex optimization problems. Therefore, this study employs this algorithm for parameter optimization in relevant models. Compared to traditional swarm intelligence optimization algorithms, SSA introduces a producer–follower–sentry population division mechanism. This ensures the algorithm’s global exploration capability while effectively enhancing local exploration precision, thereby preventing the algorithm from getting stuck in local optima. Additionally, the algorithm requires minimal parameter tuning and exhibits strong adaptive capabilities. It dynamically adjusts search stride and range during optimization, simultaneously enhancing precision and accelerating convergence. This effectively balances the algorithm’s exploration and exploitation efficiency. The novelty of this study is threefold: (1) Algorithmic—an Improved Sparrow Search Algorithm (ISSA) with Sobol initialization, opposition-based learning, adaptive Lévy flight, and hierarchical mechanisms; (2) Framework—the first integration of ISSA with XGBoost for hyperparameter optimization in leakage prediction; (3) Application field validation using real multi-sensor data from a fractured reservoir. Thus, the main innovation is the synergistic integration of these three elements. The main contributions are (i) developing ISSA to overcome SSA’s premature convergence; (ii) constructing an ISSA-XGBoost predictor; (iii) achieving 95.1% accuracy, 94.9% precision, 94.7% recall, 94.2% F1, and 4.46% AUC improvement; (iv) discussing real-field applicability such as noise robustness, early warning, false positives.

To this end, this paper first employs data preprocessing to ensure data integrity and consistency. Subsequently, by integrating Pearson correlation analysis with feature engineering methods, 11 key feature parameters highly correlated with wellbore leakage are identified. These screened sensing parameters can comprehensively reflect the structural health state and fracture evolution characteristics of underground wellbores. Building upon this foundation, an improved Sparrow Search Algorithm (ISSA) is constructed. Sobol sequences [18] are utilized for strategy initialization, while an adaptive Levy flight strategy enhances global optimization capabilities. This further optimizes the behavioral patterns of the explorer, follower, and sentinel roles through a multi-strategy role update mechanism [19]. ISSA is applied to the hyperparameter optimization process of XGBoost, to construct the ISSA-XGBoost wellbore leakage prediction model. Finally, the reliability and effectiveness of this model are validated through experiments.

## 2. Mechanism Research

### 2.1. Mechanism of Fracture-Induced Wellbore Leakage

The essence of fracture-induced wellbore leakage lies in the non-Darcy flow of drilling fluid driven by pressure differentials within natural or induced fracture networks connecting the wellbore to the formation [20]. As a typical underground engineering structure, the wellbore is susceptible to structural damage under complex in-situ stress and drilling disturbance. After the wellbore is formed, stress concentrates around the well. The density and depth of the drilling fluid primarily determine the fluid column pressure. When this pressure exceeds the formation’s fracture pressure, it induces new fractures or reopens existing ones. When the pressure lies between the pore pressure and the fracture pressure, it primarily affects the opening degree of natural fractures. After fracture initiation, drilling fluid flows into fractures driven by positive pressure differentials between the wellbore and formation. Due to the non-Newtonian nature of drilling fluids and the rough fracture walls with narrow gaps, flow follows nonlinear patterns governed by an initial pressure gradient. Loss rates exhibit complex power-law or exponential relationships with pressure differentials. Fluid loss occurs as a result of the dynamic interaction between the fluid and the fracture system. As drilling fluid wedges into deeper fracture zones, its pressure propagation alters the pressure distribution within the fractures, thereby affecting the driving pressure differential. Simultaneously, solid phases in the drilling fluid may form filter cakes or bridging within the fractures, reducing flow capacity. These physical evolution characteristics can be effectively captured by downhole monitoring sensors. Abnormal variations in sensor-monitored drilling rate and pressure parameters often indicate drilling through fractured formations, signaling a sudden increase in fluid loss risk and structural deterioration of the wellbore. Regarding practical significance, our model processes real-time multi-sensor streams with minimal latency, enabling early warning before leakage escalates. Its noise robustness ensures stable performance under field signal fluctuations, and probabilistic outputs can be integrated into existing drilling dashboards. While false positives from non-leakage events remain a challenge, our data-driven approach adapts to changing conditions better than fixed-threshold alarms. Overall, this study provides a feasible technical scheme for real-time structural health monitoring of wellbores.

### 2.2. Mechanism-Driven Feature Mapping

Based on the mechanism of fractured wellbore leakage, the key physical interactions during the leakage process are precisely correlated with quantitative indicators. These are then transformed into interpretable, computable input features to ensure the model learning process adheres to actual physical laws. The specific mapping relationships are as follows.

#### 2.2.1. Quantitative Characterization of Stress State and Crack Opening

To accurately characterize the wellbore pressure field and the integrity boundary of the wellbore wall, a quantitative characterization system is established based on drilling fluid density, well depth, and circulation pressure loss. This system enables the quantification of stress states and fracture opening, with the input layer of an improved Sparrow Search algorithm.

Equivalent circulating density (*ECD*) serves as the precise parameter for characterizing wellbore forces, comprehensively accounting for the combined effects of drilling fluid static density and circulating pressure loss. The calculation formula is given by Equation (1). Here, *ρ_m_* denotes drilling fluid density, *ΔP* represents circulating pressure loss, and *H* indicates well depth.

(1) $$ECD=\rho_{m}+\Delta P/(g\cdot H)$$

Building upon the Equivalent Circulating Density (ECD), two core indicators—overbalance pressure and fracture safety margin—are further established. The overbalance pressure reflects the excess level of wellbore fluid column pressure over formation pore pressure, while the fracture safety margin indicates the remaining capacity of the formation to resist fracturing. These two indicators are used to quantitatively assess the wellbore integrity boundary. The calculation formulas are shown in Equations (2) and (3), where $P_{over}$ represents formation pore pressure and $F_{margin}$ denotes formation fracture pressure.

(2) $${\Delta P}_{over}=ECD\cdot g\cdot H-P_{over}$$

(3) $$F_{margin}=P_{fracture}-ECD\cdot g\cdot H$$

The aforementioned quantitative parameters are coupled with the ISSA model, where the overbalance pressure and fracture safety margin are incorporated as constraints to construct the objective function, as shown in Equation (4). Here, α and β represent weighting coefficients that satisfy the condition that their sum equals 1. When *ΔP_over_* is greater than 0 and *F_margin_* is less than 0, the value of the objective function increases significantly. The ISSA model triggers a loss risk warning, indicating that the well is in a high-risk state of pressure imbalance, with the potential for a sharp increase in fracture aperture.

(4) $$min(f(x))=\alpha \cdot max(0,{\Delta P}_{over})+\beta \cdot max(0,-F_{margin})$$

#### 2.2.2. Characterization of Non-Newtonian Fluid Flow Behavior

As a typical non-Newtonian fluid, the flow resistance of drilling fluid is governed by its rheological parameters. By quantifying these rheological characteristics, a flow behavior characterization framework is established. This framework is then embedded into the fitness function optimization process of the Improved Sparrow Search Algorithm (ISSA) to enhance the prediction accuracy of fluid loss rate. Based on the Herschel–Bulkley non-Newtonian model, the relationship between the shear stress and shear rate of drilling fluid is given by Equation (5), where *τ* denotes the shear stress, *τ*_0_ refers to the yield stress, K represents the consistency coefficient, and $\gamma_{n}$ is the flow behavior index.

(5) $$\tau =\tau_{0}+K\gamma_{n}$$

Based on the shear stress relationship, the plastic viscosity is derived, which characterizes the internal friction forces of the fluid under high shear rates, as expressed in Equation (6).

(6) $$\mu_{p}=K\gamma^{n-1}$$

To distinguish the fluid flow regimes, two core dimensionless parameters are constructed—the characteristic pressure difference and the yield stress ratio. Their calculation formulas are given by Equations (7) and (8), where *ΔP* is the flow pressure differential, and v is the annular return velocity of the drilling fluid. When $\tau_{y}$ is less than 0.2, the fluid is in a linear flow regime; when it exceeds 0.2, the fluid transitions to a nonlinear flow regime. The ISSA can adaptively adjust the parameter weights of the fluid loss rate prediction model based on this criterion.

(7) $$D_{\Delta P}=\frac{\Delta P}{\rho_{m}\upsilon^{2}}$$

(8) $$D_{\tau }=\frac{\tau_{y}}{\rho_{m}\upsilon^{2}}$$

#### 2.2.3. Construction of Indicative Features for Fracture Systems and Geological Response

The development degree of fractures and the lithology of the formation are the core geological factors leading to fluid loss. By quantifying the temporal characteristics of the rate of penetration (ROP) and the formation risk level, a composite geomechanical feature vector is constructed. This serves as critical input for the ISSA to identify potential loss zones. The temporal difference in *ROP* is used as a real-time indicator of fracture development, with the magnitude of sudden *ROP* changes quantified as shown in Equation (9), where *ROP*(*t*) represents the rate of penetration at time *t*, and *ROP*(*t* − 1) represents the rate of penetration at time *t − 1*. When the current temporal difference in the rate of penetration exceeds the sum of the abrupt change threshold and the average threshold of the previous ten time points, it indicates that the drill bit may have entered a fracture-developed zone.

(9) ΔROP(t)=ROP(t)−ROP(t−1)

A composite geomechanical feature vector is constructed as shown in Equation (10) and serves as input to the ISSA, enabling the identification of potential loss zones. In Equation (10), ϕ represents porosity density and *K* denotes formation permeability and $X_{geo}$ denotes Composite Geomechanical Feature Vector.

(10) $$X_{geo}=[\Delta ROP(t),H,\phi ,K]$$

The ISSA employs a local search enhancement mechanism to iteratively optimize the feature vector, ultimately outputting a fracture development probability as expressed in Equation (11), where *σ* denotes the activation function, while *w* and *b* represent the feature weight vector and the bias term, respectively, both optimized by the ISSA algorithm.

(11) $$P_{fracture}=\sigma (w\cdot X_{geo}+b)$$

When the *fracture* development probability $P_{fracture}$ exceeds 0.7, the corresponding interval is identified as a potential loss zone, triggering the key optimization process within the ISSA.

## 3. Model Construction

### 3.1. Improving the Sparrow Search Algorithm

The Sparrow Search Algorithm (SSA) is a heuristic algorithm that mimics sparrows’ foraging and evasion behaviors. It divides the population into three functional groups—discoverers, followers, and sentinels—to explore and develop the solution space. In the parameter optimization scenario for wellbore leakage prediction, the optimization objective involves multi-parameter coupling, necessitating simultaneous consideration of multidimensional variables such as drilling pressure, drilling speed, and flow rate, resulting in an extremely high-dimensional solution space. Due to issues like abrupt changes in reservoir lithology, uneven permeability distribution, and nonlinear interactions among wellbore leakage parameters underground, the SSA algorithm often encounters multiple local optima, leading to local stagnation.

To address the SSA algorithm’s weaknesses in maintaining population diversity and low search efficiency in high-dimensional spaces, this paper proposes the Improved Sparrow Search Algorithm (ISSA) by integrating multidimensional intelligent optimization strategies. Through innovative enhancements including restructured role update mechanisms, adaptive search strategies, and strengthened global–local equilibrium capabilities, this algorithm effectively overcomes the performance limitations of the original SSA. It significantly improves optimization accuracy and robustness for complex engineering problems, providing a more reliable algorithmic framework for parameter optimization and decision-making in wellbore leakage prediction.

#### 3.1.1. Initialization Strategy for Intelligent Populations

In the iterative process of intelligent optimization algorithms, the quality of the initial population plays a decisive role in establishing the algorithm’s starting point for optimization. Traditional random initialization strategies exhibit significant flaws. Randomly generated individuals exhibit highly uneven distribution within the search space, often clustering locally. This can trap the algorithm in local optima, or a scarcity of high-quality initial individuals may substantially prolong convergence time and reduce algorithmic efficiency.

**Figure 1:**
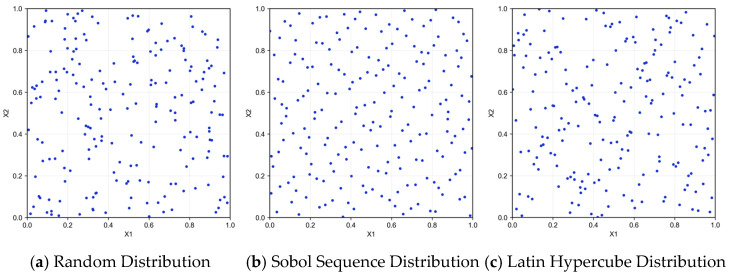
Distribution of Initial Values for Three Sequences.

Optimal point set theory provides the capability to generate uniformly distributed initial point sets, enabling individuals to cover the search space more comprehensively and enhancing population diversity. Meanwhile, the reverse learning mechanism can leverage symmetry transformations to expand individual diversity, uncovering potential high-quality solutions and providing richer initial resources for algorithmic optimization. Therefore, an intelligent population initialization strategy that integrates the strengths of both approaches has become a key direction for overcoming the limitations of traditional random initialization. Figure 1 illustrates the initial value distributions for random distribution (a), sobol sequence distribution (b), and Latin hypercube distribution (c). Comparison reveals that individuals generated based on the Sobol sequence exhibit more uniform distribution across the search space and superior quality. This provides intelligent optimization algorithms with more promising initial populations, enabling more efficient optimization processes.

The specific implementation process for generating individuals using the Sobol sequence is as follows:

(1) The *i*-th random number generated by the Sobol sequence is *S_i_* ∈ [0, 1]. The initial population position is expressed as follows:

(12) $$x_{i,j}=l_{b}+(u_{b}-l_{b})\cdot S_{i}$$

(2) For initial individuals generated from the set of optimal points, symmetric individuals are created via a reverse learning mechanism to broaden the search range. The position of a symmetric individual is represented as follows:

(13) $${\tilde{x}}_{i,j}=l_{b}+u_{b}-x_{i,j}$$

(3) For the original individual *x*_*i*,*j*_ and its reverse counterpart *x*_*i*,*j*_, the fitness function f is applied to select the superior individual and update the initial population. The selection formula is as follows:
  (14) $$x_{i,j}^{new}=\left\{\begin{array}{c} {\tilde{x}}_{i,j\;}if\;f({\tilde{x}}_{i})<f(x_{i})\\ x_{i,j}\;otherwise \end{array}\right.$$
  where $u_{b}$ and $l_{b}$ represent the upper and lower search boundaries, respectively, and $s_{i}$ denotes the optimal point set value generated by the Sobol sequence.

#### 3.1.2. Adaptive Levy Flight Search Mechanism

In the search process of intelligent optimization algorithms, balancing global exploration and local exploitation is a critical challenge. Traditional search mechanisms often suffer from insufficient exploration leading to local optima or excessive exploitation overlooking global optima due to fixed step sizes and static parameters. While Levy flight offers long-range hopping advantages, it lacks dynamic adaptability [21]. Fixed population role ratios struggle to respond to dynamic changes in the environment and objectives during search. Therefore, a mechanism integrating Levy flight characteristics with adaptive parameter control is needed to optimize search behavior and enhance algorithmic optimization efficiency.

A jump strategy was designed based on heavy-tailed distributions, using mathematical formulas to generate step sizes consistent with Levy flight characteristics. This introduces long-range exploration capabilities to the algorithm, expanding the search scope. The scale parameter *σ* is calculated using Equation (15), and the *step* size is Equation (16), where *Γ*(⋅) denotes the gamma function, *β* is the characteristic parameter of Levy flight controlling the heavy-tailed distribution shape, and *u*, *v* are normally distributed random variables.

(15) $$\sigma ={\left[\frac{\Gamma (1+\beta )\sin\left(\pi \beta /2\right)}{\Gamma ((1+\beta )/2)2^{(\beta -1)/2}}\right]}^{1/\beta }$$

(16) $$step=\frac{u}{|v{|}^{1/\beta }},\;u∼N(0,\sigma^{2}),v∼N(0,1)$$

Through adaptive parameter adjustment, dynamically optimize the ratio of explorers (*PD*) to scouts (*SD*). Design adjustment rules evolve with the iteration process, enabling population roles to adapt to the needs of each search phase. The dynamic adjustment equation for the explorer ratio is given by (17), and for the scout ratio by (18), where *t* denotes the current iteration count and *T* denotes the total iteration count.

(17) $${PD}_{adaptive}=PD\cdot \left(1+0.5\cdot \left(1-\frac{t}{T}\right)\right)$$

(18) $$SD_{adaptive}=SD\cdot (1+0.3\cdot \frac{t}{T})$$

#### 3.1.3. Multi-Strategy Role Update Mechanism

During the iterative evolution of optimization algorithms, the scientific nature of role division and update strategies directly determines the algorithm’s ability to balance exploration and exploitation. Traditional SSA algorithms, constrained by a single role-updating strategy, often face challenges in complex optimization scenarios, such as getting stuck in local optima or lacking population diversity. For instance, long-term dominance of high-quality regions by explorers leads to premature search termination, blind replication by followers causes population homogenization, and delayed response mechanisms of sentinels hinder effective escape from local optima. To overcome these limitations, the enhanced Sparrow Search Algorithm focuses on three core roles—Explorers, Followers, and Watchers, and reconstructs update rules across multiple dimensions. Through strategic coordination, it achieves the optimization objectives of more thorough global exploration, more precise local development, and sustained population vitality.

The calculated Levy flight and adaptive step size dynamically adjust explorer positions according to the following update equation:

(19) $$X_{ij}^{t+1}=\left\{\begin{array}{c} X_{ij}^{t}+step\cdot {Levy}_{j}\cdot (u_{b_{j}}-l_{b_{j}})\;if\;rand<0.5\\ X_{ij}^{t}\cdot exp\left(-i\bar{\alpha }\cdot {iter}_{max}+\epsilon \right)\;otherwise \end{array}\right.$$

The follower update incorporates crossover operations and optimal solution guidance, enabling it to maintain population diversity while following the optimal explorer. Its update equation is as follows:

(20) $$X_{ij}^{t+1}=\left\{\begin{array}{c} \;X_{bestj\;}^{t}\;if\;rand<CR\\ X_{ij\;}^{t}\;otherwise \end{array}\right.$$

The sentinel mechanism integrates three strategies—Levy flight, Gaussian mutation, and inverse learning—enhancing the algorithm’s ability to escape local optima through diversified perturbations. Its position update equation is as follows:

(21) $$X_{ij}^{t+1}=\left\{\begin{array}{c} X_{bestj}^{t}+0.1\cdot {Levy}_{j}\cdot \left(u_{j}-l_{j}\right)\\ X_{bestj}^{t}+N\left(0,2^{2}\right)\cdot \left(u_{j}-l_{j}\right)\\ l_{j}+u_{j}-X_{ij}^{t} \end{array}\right.$$

where *rand* denotes the random branch, ${Levy}_{j}$ represents a random number following a Levy distribution combined with the j-dimensional feature distribution, $u_{b_{j}}$ and $l_{b_{j}}$ denote the upper and lower bounds of the j-dimensional feature, and *CR* is the crossover rate.

### 3.2. Gradient Boosting Decision Trees

**Figure 2:**
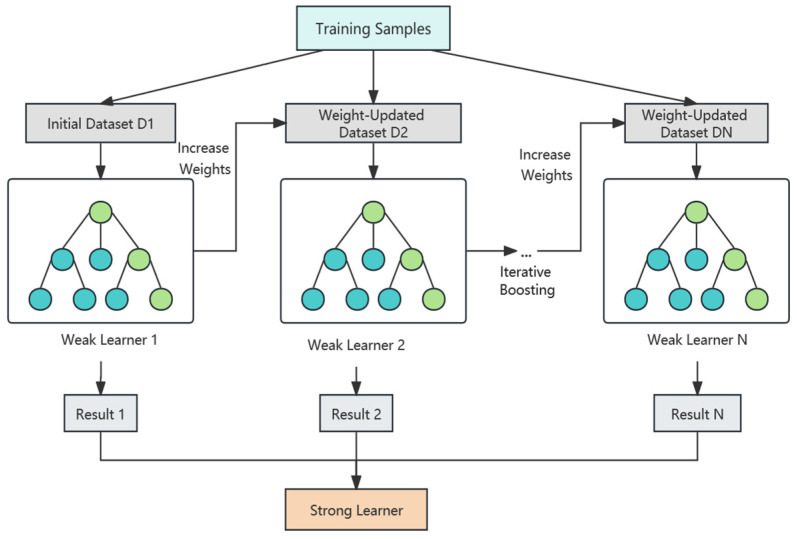
XGBoost Model Architecture Diagram.

Tree boosting is an efficient machine learning method that excels in classification and ranking tasks [22]. Extreme Gradient Boosting (XGBoost), as an efficient implementation of the tree boosting framework, follows the iterative ensemble learning paradigm to achieve precise predictions by constructing a sequence of weak learners. As illustrated in Figure 2, its core process can be decomposed as follows. First, the initial weak learner is trained using the dataset with uniform weights. Based on the prediction residuals from this learner, the sample weights are dynamically updated. Samples with larger prediction errors receive higher weights in subsequent iterations, forming a new dataset. Subsequently, subsequent weak learners are trained iteratively using a boosting mechanism. Each new learner focuses on correcting the erroneous predictions of its predecessor. Finally, the outputs of all weak learners are weighted and combined to construct a strong learner for prediction.

### 3.3. Improving Sparrow Search Algorithm for Gradient Decision Tree Optimization

**Figure 3:**
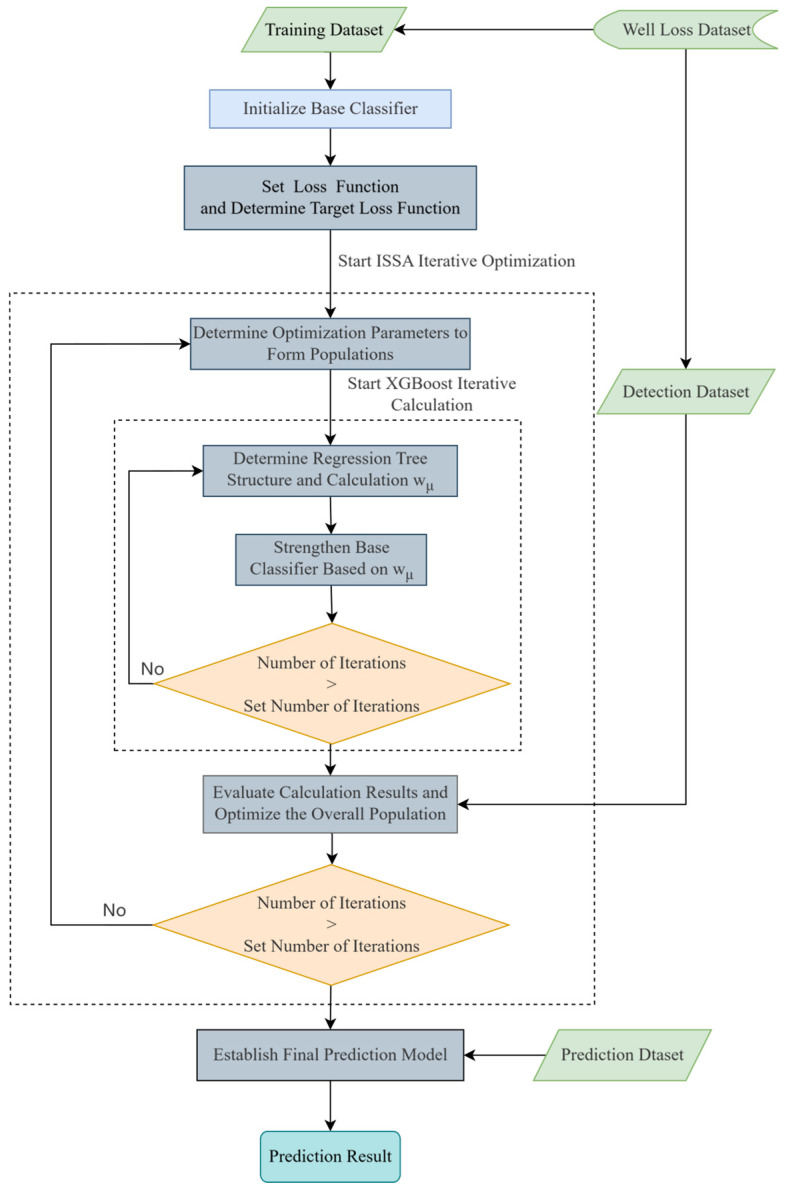
ISSA-XGBoost Model Architecture Diagram.

XGBoost incorporates numerous hyperparameters, such as tree depth (max_depth), learning rate (learning_rate), subsampling ratio (subsample), and regularization parameters (reg_alpha, reg_lambda), which play a decisive role in model performance. The ISSA-XGBoost model refers to a method that utilizes ISSA to optimize XGBoost hyperparameters, thereby enhancing model accuracy. Its principle is illustrated in Figure 3. First, data is input into XGBoost’s weak classifiers as the model foundation, defining the error metric. ISSA then simulates sparrow foraging and warning behaviors to optimize XGBoost’s hyperparameters, seeking the optimal parameter combination. ISSA encodes XGBoost parameters as population individuals, iteratively selecting the optimal parameters. The ISSA-optimized parameters are then used to train the XGBoost model, calculating the weight wμ for each regression tree. These weights adjust the training focus of the weak classifiers, iteratively strengthening the model. Iteration counts are controlled to prevent overfitting. The model’s performance is validated using a test dataset, with results fed back to ISSA to further refine the parameter population. Training iterations are adjusted to ensure parameter convergence, yielding the optimal wellbore prediction model. This model then performs inference on a prediction dataset to generate output results.

### 3.4. Step-by-Step Optimization Procedure of ISSA-XGBoost

To improve the interpretability and reproducibility of the proposed framework, the optimization procedure of ISSA-XGBoost is summarized as follows.

(1) Initialization stage:The hyperparameter search space of XGBoost is first defined, including max_depth, learning_rate, subsample, colsample_bytree, reg_alpha, and reg_lambda. The population size and maximum iteration number of ISSA are then initialized.
(2) Population initialization using Sobol sequence: Instead of random initialization, Sobol low-discrepancy sequences are employed to generate the initial sparrow population. Compared with pseudo-random sampling, Sobol sequences provide better uniformity and coverage of the search space, which improves population diversity and reduces the probability of premature convergence.
(3) Fitness evaluation: Each sparrow individual represents a candidate hyperparameter combination for XGBoost. The fitness value is calculated using prediction error metrics on the validation dataset.
(4) Position update based on Levy flight and multi-strategy mechanism: During iterative optimization, the discoverers and followers update their positions according to the improved sparrow search rules. Levy flight is introduced to enhance global exploration capability and enable the algorithm to escape local optima. Meanwhile, a multi-strategy update mechanism is adopted to dynamically balance exploration and exploitation during different optimization stages.
(5) Iterative convergence judgment: The population fitness is continuously updated until the maximum iteration number or convergence criterion is satisfied. The optimal hyperparameter combination is retained throughout the iterative process.
(6) Final model construction: The optimal hyperparameters obtained by ISSA are used to retrain the XGBoost model, thereby establishing the final prediction model for drilling parameter forecasting.

### 3.5. Rationale for the Improved Optimization Strategies

Sobol sequence initialization [23] was adopted because conventional random initialization often leads to uneven population distribution and insufficient search diversity in high-dimensional optimization problems. Sobol sequences belong to low-discrepancy sampling methods and can generate more uniformly distributed candidate solutions over the search space. Previous studies demonstrated that Sobol-based initialization significantly improves convergence speed and optimization stability in swarm intelligence algorithms.

Levy flight was introduced to strengthen the global exploration capability of SSA. Traditional SSA may suffer from premature convergence when solving complex nonlinear optimization problems. Levy flight generates occasional long-distance jumps, allowing sparrows to escape local optima and explore broader regions of the solution space. This mechanism has been widely applied in metaheuristic optimization to improve convergence accuracy and robustness.

A multi-strategy update mechanism was further employed to dynamically coordinate exploration and exploitation behaviors during optimization. In the early stage, larger search steps improve global exploration, while adaptive local refinement in later iterations enhances convergence precision. Compared with single-update strategies, multi-strategy mechanisms provide better optimization stability and prevent population stagnation.

### 3.6. Computational Complexity and Real-Time Applicability

The computational complexity of the proposed ISSA-XGBoost framework mainly depends on the population size *N*, iteration number *T*, and XGBoost training complexity. Assuming the feature dimension is *d*, the overall optimization complexity can be approximated as follows:

(22) $$O\left(T\times N\times f_{XGBoost}(d)\right)$$

where $f_{XGBoost}(d)$ denotes the computational cost of XGBoost model training.

Although the introduction of Sobol initialization, Levy flight, and multi-strategy updates slightly increases the computational overhead per iteration, these mechanisms substantially improve convergence efficiency and reduce the total number of iterations required to obtain optimal solutions. Therefore, the overall optimization cost remains acceptable for engineering applications.

In practical drilling applications, ISSA optimization is performed offline during the model training stage, whereas online prediction only requires executing the trained XGBoost model. Since XGBoost inference is computationally lightweight, the proposed framework can satisfy real-time prediction requirements in intelligent drilling systems.

## 4. Method Process

### 4.1. Multi-Source Sensing Data Acquisition and Sensing Methodology

To ensure the accuracy of wellbore leakage prediction, the datasets used in this study were collected from four vertical wells in different blocks of the Azadegan Oilfield, Iran. All wells adopted the same bottom hole assembly (BHA) and consistent drilling construction scheme, thereby avoiding equipment-induced data discrepancy. To realize real-time monitoring of downhole conditions, multiple types of high-frequency sensors were deployed to collect key drilling parameters.

Pressure sensors continuously monitor mud pressure, bottom hole pressure and casing pressure, so as to maintain downhole pressure balance and prevent hazardous incidents such as blowouts. Drilling speed sensors measure the rate of penetration to reflect real-time drilling progress. Logging-while-drilling (LWD) sensors acquire formation characteristics including lithology, density and porosity. Meanwhile, mud flow sensors track drilling fluid flow rate, to keep boreholes stable and avoid stuck pipes.

Additional sensors record weight on bit, borehole diameter, shear stress, gel strength, and chloride pressure. These parameters are vital for evaluating drilling performance and formation response. In this study, a total of 5000 valid data records were initially compiled, covering 19 raw parameters numbered Z1–Z19. The detailed statistical characteristics of these parameters are listed in Table 1.

### 4.2. Data Preprocessing

#### 4.2.1. Data Cleaning and Denoising

To enhance data quality, data cleaning was firstly conducted for the raw dataset. First, outliers that violate logical and common-sense principles were removed based on empirical rules [24]. Specifically, for each drilling parameter, we defined physical upper and lower bounds according to field operation specifications and expert experience. Any measurement exceeding these bounds was identified as an outlier and eliminated. On this basis, the remaining anomalous points were further processed adopting the 3σ statistical criterion. Data points exceeding the range of [μ − 3σ, μ + 3σ] were defined as abnormal outliers and replaced with reasonable interpolation values. This dual screening strategy combining empirical physical constraints and statistical criteria ensured rationality and comprehensive outlier discrimination.

**Figure 4:**
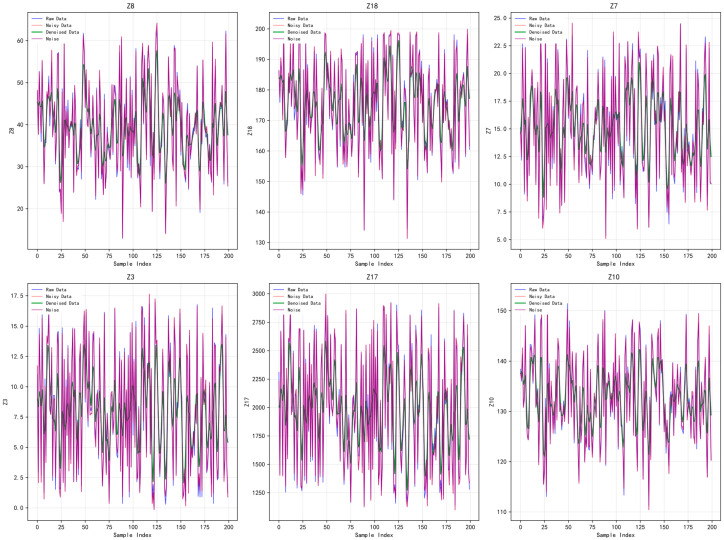
Comparison of Data Before and After Cleaning.

Second, missing values were handled using median imputation. The median imputation is mathematically expressed as follows:

(23) $$x_{imputed}=median\left(X_{valid}\right)$$

where $x_{imputed}$ denotes the set of observed valid values for the corresponding parameter. This method fills each missing entry with the median of the available non-missing data for that parameter. To obtain higher-quality data, noise addition and denoising operations were performed. The noise addition better validated the effectiveness of the denoising algorithm. Figure 4 presents a comparison of selected features between the original data and the denoised version. The denoised data exhibits a trend highly consistent with the original records, indicating that noise interference has been effectively suppressed. To obtain higher-quality data, Gaussian White Noise addition and Moving Average denoising operations were further performed in this study. To evaluate the robustness of the denoising method, we first added controlled synthetic noise to the cleaned raw data. The added noise followed a zero-mean Gaussian distribution, with its standard deviation set to 5% of the mean value of each corresponding drilling parameter. This configuration simulates the typical range of random sensor noise encountered in field drilling environments, creating a realistic test scenario for denoising validation. The noise addition better validated the effectiveness of the denoising algorithm. A simple moving average filtering method with a window size of five sampling points was utilized to smooth raw data. The filtering process eliminates random high-frequency interference while retaining effective trend variation of drilling parameters. Figure 4 presents a comparison of selected features between the original data and the denoised version. The denoised data exhibits a trend highly consistent with the original records, indicating that noise interference has been effectively suppressed.

#### 4.2.2. Feature Extraction

Traditional wellbore leakage prediction methods often lack systematic parameter selection, and different parameters exert varying influences on leakage prediction. Since data correlations affect model training speed and effectiveness, input features must be appropriately selected prior to model training.

**Figure 5:**
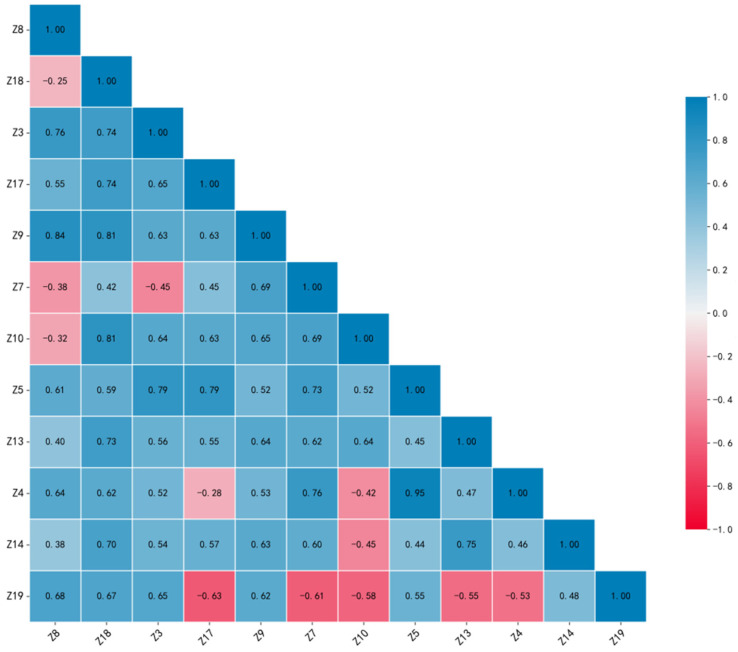
Heatmap of Pearson Correlation Coefficients.

This experiment first employed a Pearson correlation analysis model [25] to evaluate the linear correlation among features, selecting the 11 most highly correlated feature parameters. Their correlation coefficients are shown in Figure 5. The Pearson correlation coefficient r ranges [−1, 1], where a positive r indicates positive correlation and a negative r indicates negative correlation.

However, lower correlations among other parameters do not imply their insignificance in influencing well leakage. These parameters may affect leakage occurrence through interactions with other factors or via indirect mechanisms. Therefore, we further employed feature importance analysis based on the ISSA-XGBoost model, Spearman’s correlation coefficient [26], and mutual information [27] to explore nonlinear relationships. The model feature importance method quantifies each feature’s contribution to prediction accuracy by calculating the reduction in Gini coefficient during decision splitting, with values ranging from −1 to 1. The Spearman correlation coefficient measures monotonic nonlinear relationships between two variables, with values ranging from 0 to 1. Mutual information captures arbitrary dependencies between features, encompassing both linear and nonlinear relationships, with values ranging from [0, 1]. A value of 0 indicates complete independence, while 1 signifies perfect correlation.

**Figure 6:**
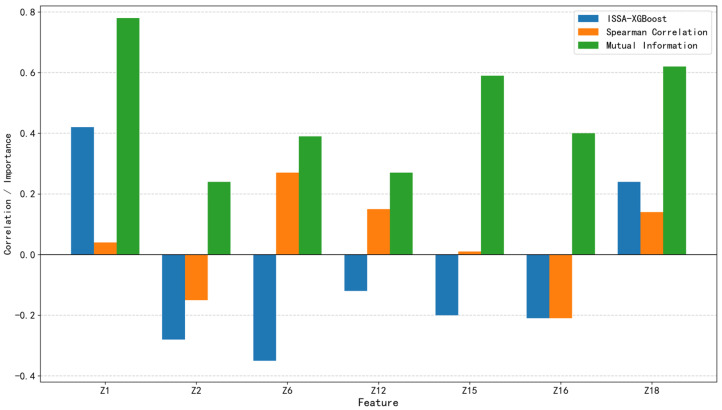
Comparison of nonlinear relationships between well leakage and key features.

Figure 6 compares the correlation coefficients and feature importance scores between well leakage and key characteristics obtained from three methods: the ISSA-XGBoost model, Spearman correlation analysis, and mutual information. The results reveal significant discrepancies across different approaches, reflecting the diverse relationships between features and well leakage. Specifically, Feature Z1 exhibits the highest importance in both the ISSA-XGBoost model and mutual information, while its Spearman correlation coefficient is only 0.045, indicating a strong nonlinear association with well leakage rather than a simple linear relationship. In contrast, Feature Z6 shows contradictory trends: a negative contribution in the ISSA-XGBoost model but a positive linear correlation in Spearman analysis, suggesting potential complex interactions or confounding effects with other variables. Feature Z18 demonstrates consistent positive correlations across all three methods, with a relatively high mutual information value, indicating a stable positive association with well leakage. Overall, mutual information effectively captures nonlinear dependencies, while the ISSA-XGBoost model prioritizes features with both linear and nonlinear predictive power. These findings highlight that relying solely on linear correlation analysis may overlook critical predictive features, emphasizing the necessity of combining multiple methods for robust feature selection in well leakage prediction modeling.

Based on the above analysis, this study ultimately selected these 18 parameters as feature variables for the model.

#### 4.2.3. Handling Imbalanced Categories

**Figure 7:**
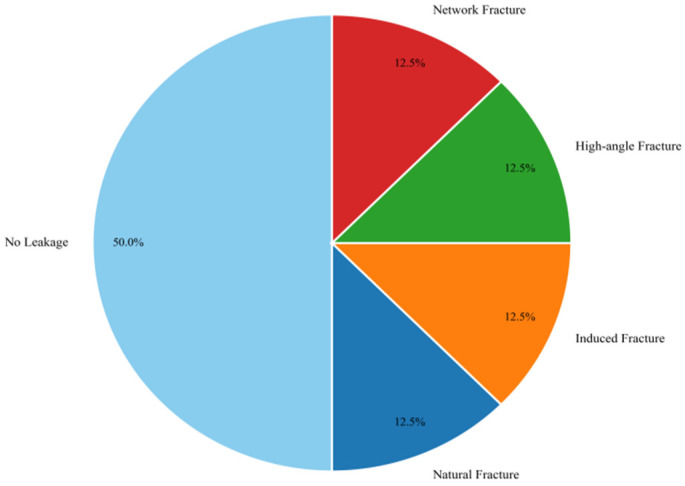
Data Distribution Chart.

During drilling operations, wellbore leakage exhibits significant regional variations. In areas with complex geological structures, leakage frequently occurs and may even become a routine occurrence during drilling. However, in regions with relatively simple geological conditions, leakage remains a high-risk and relatively rare drilling incident. To accommodate these regional differences, the collected dataset underwent class imbalance treatment. The original dataset, collected from the Azadegan oilfield, contained 5000 samples with mild class imbalance; 3000 samples (60.0%) belonged to the majority “No Leakage” class, while 2000 samples (40.0%) were leakage cases. Among these leakage samples, natural fracture loss, induced fracture loss, high-angle fracture loss, and network fracture loss accounted for 550 (11.0%), 520 (10.4%), 490 (9.8%), and 440 (8.8%) samples, respectively. Such mild imbalance may lead to slight prediction bias and weaken the model recognition performance for various leakage types. Given the strong coupling relationships among drilling parameter features, the SMOTE hybrid sampling [28] method was employed to better preserve the physical correlations between parameters. Figure 7 illustrates the data distribution after class imbalance correction, comprising 2500 normal data points and 2500 abnormal data points and each leakage type occupied an equal proportion in the whole dataset. The abnormal points are further categorized by loss type: natural fracture loss, induced fracture loss, high-angle fracture loss, and network fracture loss. The processed dataset was divided into training and test sets. After random shuffling of the entire dataset, 70% and 30% were allocated for training and testing, respectively. Despite the effective alleviation of class imbalance using the SMOTE algorithm, it is necessary to discuss its potential overfitting risk. Blind synthetic sampling may generate ambiguous boundary samples and introduce artificial noise, which reduces the model generalization ability and causes minor overfitting. To mitigate this risk, several constraint strategies were adopted in this study. First, the SMOTE sampling process was only performed on the training set, while the independent test set remained unchanged to strictly avoid data leakage. Second, a moderate k-nearest neighbor parameter (k = 5) was selected to prevent excessive interpolation and sample aliasing. Third, cross-validation was utilized to monitor the training deviation. The above methods effectively limit the overfitting risk caused by synthetic samples, ensuring that the generated minority samples conform to the actual distribution of downhole leakage characteristics. Therefore, the adopted SMOTE strategy remains reasonable and reliable for imbalanced drilling data classification.

#### 4.2.4. Data Normalization

Data normalization is crucial for model training. Logging data from drilling operations exhibits a wide numerical range and requires scaling to a common range to accelerate the learning process. Furthermore, normalization accelerates convergence when training models using gradient-based optimization algorithms, enabling models to find optimal solutions more rapidly. Therefore, the Min–Max Scaling method [29] was employed to scale input sequences to the 0–1 range; *x_i_* represents the original data, and *y*_i_ denotes the normalized data, as expressed in Equation (24):

(24) $$y_{i}=\frac{\left(x_{i}-\bar{x}\right)}{\left(x_{max}-x_{min}\right)}$$

### 4.3. Model Evaluation

In this study, to comprehensively evaluate prediction results, we selected accuracy (the proportion of correct predictions among all predictions), precision (the proportion of actual positive samples among those predicted as positive), recall (the proportion of correctly predicted positive samples among all positive samples), and F1 score (a harmonic mean of precision and recall, comprehensively evaluating both performance metrics). Recall (the proportion of actual positive samples correctly predicted), and F1 score (a harmonic mean of precision and recall, comprehensively evaluating both metrics) as evaluation indicators. When evaluating the predictive performance of machine learning models, accuracy, precision, recall, and F1 score are highly common key metrics. They enable us to deeply analyze the model’s performance across various scenarios, playing a crucial role especially when dealing with class imbalance or situations where misclassification of different categories incurs varying costs. Their specific calculation formulas are as follows. In the formula, *TP* denotes a true positive, where both the actual classification and the model prediction are positive; *TN* denotes a true negative, where both the actual classification and the model prediction are negative; *FP* denotes a false positive, where the actual classification is negative but the model prediction is positive; *FN* denotes a false negative, where the actual classification is positive but the model prediction is negative.

(25) $$Accuracy=\frac{TP+TN}{TP+FP+TN+FN}$$

(26) $$Precision=\frac{TP}{TP+FP}$$

(27) $$Recall=\frac{TP}{TP+FN}$$

(28) $$F1=\frac{2\times Recall\times Precision}{Recall+Precision}$$

## 5. Experimental Results and Discussion

### 5.1. Hyperparameter Optimization

All experiments were conducted on a Windows system equipped with an Intel Core i7-12400 CPU, 32 GB RAM, and an NVIDIA GeForce RTX 4060 Ti 16GB. Development utilized Python 3.10 and TensorFlow 2.10.0.

To maximize model accuracy, random search was employed to identify key hyperparameters. The optimized hyperparameters included the number of trees (n_estimators), maximum tree depth (max_depth), learning rate (learning_rate), subsample ratio (subsample), colsample_bytree ratio (colsample_bytree), and L1 regularization (reg_alpha). A Sobol sequence was used to extract 100 mutually exclusive parameter combinations from the initial dataset. An ISSA-XGBoost model was constructed for each combination, trained on a unified training set, and validated on the test set. To avoid bias toward imbalanced samples from a single accuracy metric, the F1 score was selected as the comprehensive performance evaluation metric. Relevant literature was consulted to determine approximate parameter ranges, as shown in Table 2. The search ran for 50 iterations to ensure full convergence.

Through the parameter search, the optimal configuration was identified at Epoch = 50:300 trees, maximum tree depth of 7, learning rate of 0.08, subsampling ratio of 0.85, feature sampling ratio of 0.9, and L1 regularization of 0.05.

### 5.2. Ablation Experiments

To rigorously evaluate the independent contributions and synergistic effects of the three key improvement strategies proposed in this paper—Intelligent Population Initialization Strategy (IPIS), Adaptive Levy Flight Search Mechanism (ALF), and Multi-Strategy Role Update Mechanism (MSRU)—on the performance of the ISSA algorithm, this study designed systematic ablation experiments. Experiments were conducted by constructing eight algorithm variants and testing them on the high-dimensional unimodal function F1, the high-dimensional multimodal function F2, and the low-dimensional multimodal function F3. The single-peak function possesses only one optimal solution, serving to evaluate the algorithms’ convergence speed. The multi-peak function exhibits multiple local optima, a characteristic that readily traps algorithms in local optima. Therefore, the algorithms’ performance in escaping local optima was tested from both high-dimensional and low-dimensional perspectives, with the standard deviation shown in Table 3. Based on the ablation results, the inherent superiority of ISSA can be explicitly explained from the perspective of algorithm optimization logic. Specifically, each improved module performs a clear and independent function and jointly enhances the comprehensive optimization capability. The IPIS adopts the Sobol sequence to generate uniformly distributed initial populations, avoiding individual aggregation and expanding the early search coverage; the ALF introduces variable step-size flight characteristics to strengthen global exploration ability and effectively alleviate the local optimum dilemma; the MSRU optimizes the hierarchical position update rules for producers and scroungers, reducing iterative oscillation and improving convergence stability. Notably, the proposed improvements introduce only negligible additional computational overhead compared to the original SSA. The Sobol sequence initialization is executed once before iteration, with a time complexity of O (N log N), which is far lower than the iterative optimization cost. The adaptive Levy flight and multi-strategy update mechanisms only add lightweight conditional judgments and mathematical operations in each iteration, without significantly increasing the per-iteration computation time. Meanwhile, ISSA achieves faster convergence speed than SSA, requiring fewer iterations to converge to the optimal solution. Therefore, the overall time complexity of ISSA remains at the same level as the original SSA, avoiding the common problem of performance improvement at the cost of excessive computational burden. Benefiting from the above collaborative optimization strategies, ISSA achieves faster convergence speed and more accurate hyperparameter searching capability. When embedded into the XGBoost framework, the optimized hyperparameters effectively reduce prediction errors, thereby enabling the ISSA-XGBoost to obtain stable and superior prediction performance compared with other conventional machine learning models.

As shown in the table, compared to the original SSA, the three variants incorporating a single improvement strategy demonstrated significant improvements in optimization results across all three test functions. This indicates that each individual mechanism, including the Sobol sequence initialization, adaptive Lévy flight, and multi-strategy role update, can effectively enhance the search capability of the algorithm from different aspects. The dual-strategy variants generally outperform any single-strategy variant, confirming positive synergies among the improved components. Specifically, the combination of uniform population initialization and adaptive flight behavior effectively balances early exploration and later exploitation, while the hierarchical role update further stabilizes the convergence process. The ISSA algorithm incorporating all three strategies achieved the highest optimization accuracy across all test functions. This comprehensive improvement enables ISSA to achieve faster convergence, stronger global search ability, and higher solution precision compared to both the original SSA and its single- or dual-strategy variants.

### 5.3. ISSA Performance Optimization Analysis

**Figure 8:**
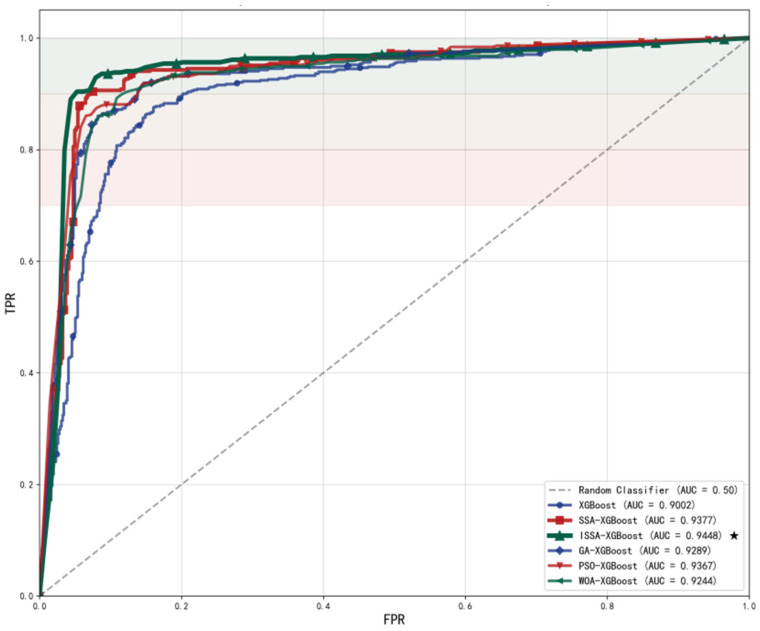
AUC Values for Different Optimization Algorithms. The background color bands (green, orange, red) indicate high, moderate, and low performance ranges, respectively. The star symbol (★) denotes the proposed ISSA-XGBoost model, which obtained the best AUC value of 0.9448.

To deeply analyze the mechanism by which improvements to the Sparrow Search Algorithm enhance optimization performance, this study focuses on the core enhancement modules of the algorithm. By comparing the Standard Sparrow Algorithm (SSA), Genetic Algorithm (GA), Particle Swarm Optimization (PSO), and Whale Optimization Algorithm (WOA), which are classic intelligent optimization algorithms, the optimization performance of the Improved Sparrow Search Algorithm (ISSA) was validated. The results are shown in Figure 8.

As shown in the figure, the ISSA-optimized XGBoost model demonstrated the best performance, achieving an AUC value of 0.9448. This was significantly higher than the original XGBoost and its improved variants—SSA-XGBoost, PSO-XGBoost, and WOA-XGBoost. All XGBoost models enhanced with optimization algorithms demonstrated superior AUC compared to the original XGBoost, indicating that optimization algorithms can effectively enhance XGBoost classification performance. Among these, the ISSA optimization strategy stands out for its ability to enhance model classification accuracy while balancing true positive rate and false positive rate, making it particularly suitable for scenarios demanding high classification accuracy.

### 5.4. Comparative Experiments

To comprehensively evaluate the performance of the ISSA-XGBoost model in wellbore leakage prediction, a comparative experiment was conducted against classic leakage prediction models: Random Forest (RF), Gradient Boosting Decision Tree (GBDT), Support Vector Machine (SVM), Long Short-Term Memory (LSTM), and Light Gradient Boosting (Light GBM). The specific workflow involved first using random search to determine the optimal parameter combinations for the three comparison models. Subsequently, wellbore leakage prediction experiments were conducted under identical parameter configurations to compare the predictive efficacy of each model.

**Figure 9:**
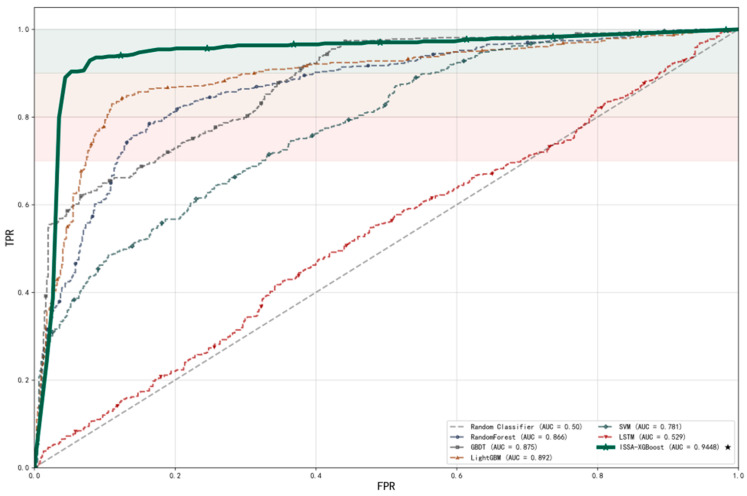
Comparison of ROC Curves and AUC Values. The background color bands (green, orange, red) indicate high, moderate, and low performance ranges, respectively. The star symbol (★) denotes the proposed ISSA-XGBoost model, which obtained the best AUC value of 0.9448.

Figure 9 displays the ROC curves and AUC values of the ISSA-XGBoost model compared to other models. The ISSA-XGBoost model demonstrated the highest AUC values, outperforming common wellbore leakage prediction models. Furthermore, the ISSA-XGBoost ROC curve lies closer to the top-left corner across the entire FPR range. This indicates that the ISSA-XGBoost model exhibits higher accuracy in addressing wellbore leakage prediction problems, delivering superior performance with outstanding classification capabilities and the lowest risk of misclassification.

**Figure 10:**
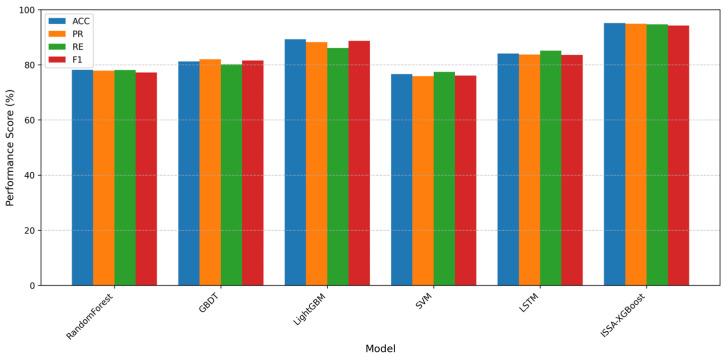
Accuracy Distribution Chart for Different Models.

As shown in Figure 10, in the evaluation of wellbore leakage detection, the ISSA-XGBoost model achieved the best performance, with an ACC of 95.1%, precision of 94.9%, recall of 94.7%, and F1 score of 94.2%. As key metrics for evaluating classificationperformance, higher values of ACC, precision, recall, and F1 score indicate stronger model capabilities in accurately identifying wellbore leakage and non-leakage samples. The ISSA-XGBoost model significantly outperformed all baseline models (RandomForest, GBDT, LightGBM, SVM, and LSTM), demonstrating superior classification efficacy in wellbore leakage detection tasks. It accurately identifies leakage samples and effectively reduces false alarms, providing reliable support for early warning of wellbore leakage risks. This model thus shows outstanding advantages in leakage prediction applications under complex drilling conditions.

Table 4 presents the evaluation metrics for different models. These data indicate that the ISSA-XGBoost model demonstrates higher accuracy in wellbore leakage prediction. The following table shows the evaluation metrics for the prediction results of various models. As evident from the data, the ISSA-XGBoost model also outperformed other models in precision, recall, and F1 score metrics, exhibiting the highest overall performance.

To further interpret the model’s decision-making process, we analyzed the feature importance scores derived from the trained XGBoost model. The results show that drilling fluid loss rate, pump pressure, and rate of penetration are the top three contributing features, which align well with the physical mechanism of fractured leakage. This analysis provides practical guidance for field engineers to focus on these key parameters when monitoring and preventing wellbore leakage.

### 5.5. Model Generalization Validation

**Figure 11:**
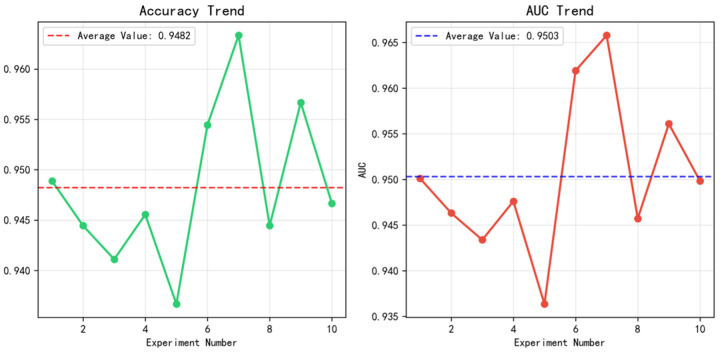
Trend Chart of Accuracy and AUC for Other Wells.

To validate the generalization capability of the ISSA-XGBoost model, experiments were conducted on two wells (Well B and Well C) from different regions. Each well underwent five experimental sets (Experiments 1–5 corresponded to Well B data, while Experiments 6–10 corresponded to Well C data), with metrics shown in Figure 11. Based on the trends in accuracy and AUC results, the average accuracy reached 0.9482, with an average AUC of 0.9503. Both metrics remained at high levels with relatively stable fluctuations. This demonstrates that the ISSA-XGBoost model can effectively adapt to variations in data distribution across different well conditions in diverse regions, exhibiting strong generalization performance. It provides robust data support and experimental evidence for the model’s practical application in scenarios such as wellbore leakage prediction, validating its potential to maintain stable predictive capabilities in complex geological environments.

**Figure 12:**
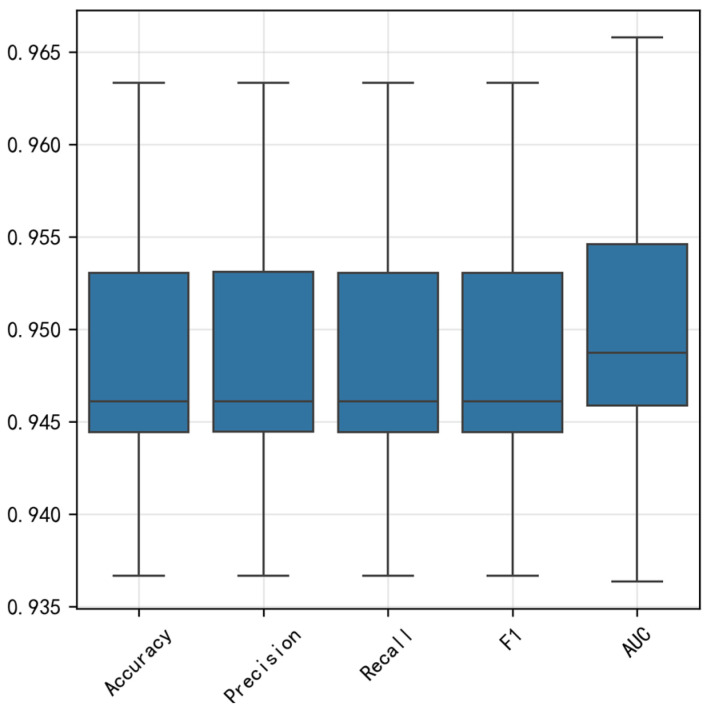
Box Plot of Performance Metric Distribution.

Figure 12 presents box plots of key performance metrics after ten model experiments, covering accuracy, precision, recall, F1 score, and AUC. The box plots visually illustrate the distribution characteristics of each metric. As shown, the boxes for all metrics cluster within higher numerical ranges, indicating stable model output across multiple experiments. Specifically, the boxes for accuracy, precision, recall, and F1 score are compact with substantial median values, while the AUC metric also maintains a favorable level. This reflects the model’s outstanding capability in distinguishing positive and negative samples, achieving high precision and comprehensiveness in category prediction during classification tasks. The overall stable classification performance effectively validates the model’s reliability and effectiveness in practical application scenarios.

### 5.6. Further Discussion and Statistical Analysis

To further explain the internal optimization mechanism of the ISSA-XGBoost model, the essential reasons for its superior performance are systematically analyzed in this section. Firstly, the improved sparrow search algorithm optimizes the initial hyperparameters of XGBoost, avoiding local optimum stagnation caused by artificial parameter tuning. Secondly, the adaptive weight update strategy enhances the population diversity of sparrow individuals, which strengthens the global searching ability for leakage feature thresholds. Thirdly, the anti-collapse mechanism effectively reduces sensitivity to noisy drilling data, thereby improving prediction stability. To verify the statistical significance of model differences, the paired t-test was conducted among all comparative models. The results demonstrate that ISSA-XGBoost achieved statistically significant differences (*p* < 0.05) in evaluation metrics compared with the other machine learning methods, proving that the performance improvement is not accidental.

Furthermore, the computational complexity and runtime consumption of each model are supplemented. The lightweight tree structure of XGBoost ensures low computational overhead, and the ISSA optimization only consumes limited iterative resources. The average inference time of the proposed model is less than 0.03 s, which fully meets the low-latency requirement of field drilling monitoring. In terms of noise robustness, multiple groups of Gaussian noise with different intensities were added into raw drilling data. The results show that ISSA-XGBoost maintains stable accuracy under noisy interference, indicating strong anti-noise robustness for complex downhole working conditions.

## 6. Summary

Wellbore leakage in fractured formations arises from the complex coupling of multiple physical and geological factors, where dynamic pressure balance among fluid pressure, pore pressure, and fracture pressure is critical for downhole structural stability. Most existing data-driven leakage models capture only shallow statistical correlations from monitoring data and lack explicit consideration of underlying physical mechanisms, leading to limited generalization and prediction reliability.

To address this gap, this study proposes an ISSA-XGBoost intelligent prediction framework for fractured wellbore leakage identification. The improved sparrow search algorithm (ISSA) integrates several strategies to optimize the hyperparameters of the XGBoost model, including Sobol sequence for uniform population initialization, opposition-based learning to enhance diversity, adaptive Lévy flight to improve global search, and crossover operations with a hierarchical leader–follower mechanism to refine follower movements. Based on multi-sensor data collected from a typical fractured reservoir block in the Azadegan Oilfield (Iran), the proposed model achieved an AUC improvement of 4.46%, along with accuracy (95.1%), precision (94.9%), recall (94.7%), and F1 score (94.2%). Comparative results demonstrate that ISSA-XGBoost significantly outperforms conventional algorithms such as LSTM, random forest, and SVM, confirming its strong generalization ability and practical applicability for real-time structural health monitoring of downhole wellbore structures.

In summary, this study validates the effectiveness of combining multi-sensor monitoring data with an intelligent optimization method for wellbore structural hazard prediction, providing a feasible technical scheme for leakage risk prevention in petroleum drilling engineering. Although the current work focuses on fractured leakage scenarios in the Azadegan field, the proposed framework is inherently flexible and data-driven, relying on commonly monitored drilling parameters rather than fracture-specific assumptions. Therefore, it has the potential to be extended to other leakage mechanisms such as vuggy or permeable losses, provided that sufficient representative data from these scenarios are incorporated into the training set. Nevertheless, under complex downhole conditions, non-leakage abnormal events may cause similar fluctuation characteristics in sensor data, triggering false positive warnings. Future research will focus on enhancing anti-interference capability through multi-source data fusion, reducing false alarms, and extending the proposed framework to comprehensive drilling risk management and long-term structural health perception.

## Figures and Tables

**Table 1:** Well Leakage Prediction Dataset Feature Table.

Number	Well Leakage Parameter	Units	Minimum Value	Maximum Value
Z1	North Coordinate	m	1,887,146	1,936,035
Z2	East Coordinate	m	1,005,840	1,049,982
Z3	Depth	m	0	500
Z4	Bit Depth	m	0.22	650
Z5	Drilling Time	h	0.1	24
Z6	Formation Type	-	1	6
Z7	Hole Diameter	Inch	4.125	26
Z8	Weight on Bit	b	1	70
Z9	Flow Rate	Gpm	80	1700
Z10	Density	Pcf	30.5	160.5
Z11	Marsh Funnel Viscosity	-	27	100
Z12	Solid Content	%	0	61
Z13	Pore Pressure	Psi	1.255	10,508
Z14	Fracture Pressure	Psi	1.971	13,609
Z15	Shear Stress (600/300)	-	1.21	196
Z16	Gel Strength (10 min_10 s)	-	1.11	2.89
Z17	Chloride Pressure	Psi	296.9	2954.1
Z18	Drilling Speed	RPM	42.82	198.1
Z19	Target	-	0	1

**Table 2:** Hyperparameter Search Range.

Hyperparameter Name	Search Scope
n_estimators	[56, 485]
max_depth	[3, 9]
learning_rate	[0.0264, 0.2800]
subsample	[0.60, 0.96]
colsample_bytree	[0.6, 1]
reg_alpha	[0.891, 0.979]

**Table 3:** Comparison of Optimization Performance for Different Algorithm Variants on Test Functions.

Algorithm Function	F1(x)	F2(x)	F3(x)
SSA	3.45 × 10^−2^	2.89 × 10^1^	1.56 × 10^−1^
SSA-IPIS	1.89 × 10^−2^	2.12 × 10^1^	1.21 × 10^−1^
SSA-ALF	2.76 × 10^−2^	2.45 × 10^1^	1.42 × 10^−1^
SSA-MSRU	2.01 × 10^−2^	2.30 × 10^1^	1.38 × 10^−1^
SSA-IPIS-ALF	9.87 × 10^−3^	1.78 × 10^1^	8.76 × 10^−2^
SSA-IPIS-MSRU	8.54 × 10^−3^	1.65 × 10^1^	7.89 × 10^−2^
SSA-ALF-MSRU	7.21 × 10^−3^	1.42 × 10^1^	6.54 × 10^−2^
ISSA	4.32 × 10^−3^	8.76 × 10^0^	3.21 × 10^−2^

**Table 4:** Evaluation Metrics for Different Models.

Model	ACC	PR	RE	F1
Random Forest	78.2%	77.9%	78.1%	77.2%
GBDT	81.2%	82.0%	80.2%	81.6%
LightGBM	89.3%	88.2%	86.1%	88.7%
SVM	76.6%	75.9%	77.4%	76.1%
LSTM	84.1%	83.7%	85.1%	83.6%
ISSA-XGBoost	95.1%	94.9%	94.7%	94.2%

## Data Availability

The dataset used in this study is not publicly available. Researchers with requests should contact the corresponding author. We will provide support and additional information within reasonable boundaries.
